# Telomere Length and the Risk of Cutaneous Malignant Melanoma in Melanoma-Prone Families with and without *CDKN2A* Mutations

**DOI:** 10.1371/journal.pone.0071121

**Published:** 2013-08-19

**Authors:** Laura S. Burke, Paula L. Hyland, Ruth M. Pfeiffer, Jennifer Prescott, William Wheeler, Lisa Mirabello, Sharon A. Savage, Laurie Burdette, Meredith Yeager, Stephen Chanock, Immaculata De Vivo, Margaret A. Tucker, Alisa M. Goldstein, Xiaohong R. Yang

**Affiliations:** 1 Division of Cancer Epidemiology and Genetics, National Cancer Institute, National Institutes of Health, Department of Health and Human Services, Rockville, Maryland, United States of America; 2 Cancer Prevention Fellowship Program, Office of Directors, National Cancer Institute, National Institutes of Health, Department of Health and Human Services, Rockville, Maryland, United States of America; 3 Department of Epidemiology, Harvard School of Public Health, Boston, Massachusetts, United States of America; 4 Program in Molecular and Genetic Epidemiology, Harvard School of Public Health, Boston, Massachusetts, United States of America; 5 Channing Laboratory, Department of Medicine, Brigham and Women’s Hospital and Harvard Medical School, Boston, Massachusetts, United States of America; 6 Information Management Services, Inc., Rockville, Maryland, United States of America; 7 Core Genotyping Facility, SAIC-Frederick, Inc., National Cancer Institute -Frederick, Frederick, Maryland, United States of America; The University of Queensland, Australia

## Abstract

**Introduction:**

Recent evidence suggests a link between constitutional telomere length (TL) and cancer risk. Previous studies have suggested that longer telomeres were associated with an increased risk of melanoma and larger size and number of nevi. The goal of this study was to examine whether TL modified the risk of melanoma in melanoma-prone families with and without *CDKN2A* germline mutations.

**Materials and Methods:**

We measured TL in blood DNA in 119 cutaneous malignant melanoma (CMM) cases and 208 unaffected individuals. We also genotyped 13 tagging SNPs in *TERT*.

**Results:**

We found that longer telomeres were associated with an increased risk of CMM (adjusted OR = 2.81, 95% CI = 1.02–7.72, *P* = 0.04). The association of longer TL with CMM risk was seen in *CDKN2A*- cases but not in *CDKN2A*+ cases. Among CMM cases, the presence of solar injury was associated with shorter telomeres (*P* = 0.002). One SNP in *TERT*, rs2735940, was significantly associated with TL (*P* = 0.002) after Bonferroni correction.

**Discussion:**

Our findings suggest that TL regulation could be variable by *CDKN2A* mutation status, sun exposure, and pigmentation phenotype. Therefore, TL measurement alone may not be a good marker for predicting CMM risk.

## Introduction

Cutaneous malignant melanoma (CMM) is an etiologically heterogeneous disease with genetic, host, environmental factors, and their interactions contributing to its development. The main environmental risk factor is ultraviolet radiation (UVR), which may influence melanoma risk through multiple mechanisms, such as directly causing DNA damage, influencing the expression of apoptosis-related molecules and inducing immunosuppression. [1] Host phenotypic factors such as having a large number of benign and dysplastic nevi (DN), blond or red hair color, light eye color, freckling, and poor tanning ability have also been associated with increased melanoma risk. [2] Approximately 10% of CMM cases occur in a familial setting. [3] To date, two high-risk melanoma susceptibility genes, *CDKN2A* on chromosome 9p21 and *CDK4* on 12q14, have been identified. Germline mutations of the *CDKN2A* gene have been described in approximately 20% of familial melanoma kindreds. [4]–[5] Mutations of *CDK4* are rare, and only a few families worldwide have been found to harbor mutations. Although germline *CDKN2A* mutations are associated with a high risk of CMM, the penetrance of this gene is incomplete and varies by age and geographical location. [6] Additionally, phenotypic manifestations such as age at diagnosis, presence/number of DN, number of melanomas, and cosegregation of pancreatic cancer vary significantly among mutation carriers even within a single family. These findings suggest that other factors modify the effect of *CDKN2A*.

Telomeres are located at the ends of chromosomes, and consist of tandem nucleotide repeats (TTAGGG)_n,_ the telomerase enzyme, the shelterin protein complex, and many other accessory proteins. They maintain genomic stability and chromosomal integrity by protecting chromosome ends from degradation, end-to-end fusion, and atypical recombination. [7] Telomeres shorten with each cell division, due to ineffective replication of the 3′ end of DNA. [8] The telomerase enzyme complex consists of the reverse transcriptase, *TERT*, and additional proteins which are essential to maintain telomere length (TL). Telomerase is upregulated in the majority of cancers [9] and in the immortalization of skin keratinocytes. [10] Previous studies have shown that genetic variation in *TERT* was associated with melanoma risk. [11] Recently, a germline mutation in the promoter of *TERT* was identified in a melanoma-prone family that caused a 2–4 fold increase of *TERT* transcription. [12] Multiple mutations in the *TERT* promoter were also found in primary melanoma tissues with high frequency (33%),[12]–[13] suggesting that the dysregulation of *TERT* may play an important role in the genesis of melanoma.

TL is influenced by multiple factors, including both genetic and environmental. A twin study indicated 78% heritability for mean TL in blood [14], and subsequent studies mapped several candidate loci for TL using linkage and genome-wide association analyses. [15]–[20] Telomeres are highly sensitive to damage by oxidative stress, alkylation, and UVR, which can cause telomere shortening without DNA replication by inducing telomeric double-strand breaks at high frequency. [21] Epidemiologic studies examining the association between constitutional TL and cancer risk have generated inconsistent results. Although shortened TL has been associated with increased risk of a number of cancers such as bladder, gastric, and head and neck [22], associations between longer TL and increased risk were reported for other cancer types including Non-Hodgkin Lymphoma [23], Hepatitis B Virus-Related Hepatocellular Carcinoma [24], and melanoma. [25]–[26] Similarly, a previous study also found that longer telomeres were associated with larger size and number of melanocyte nevi. [27] These findings suggest that the association of TL with cancer risk is complex and cancer-type specific, which can be tumor suppressing or promoting depending on the host or cell type’s susceptibility to genetic and environmental exposures. The goal of our study was to examine whether TL modified the risk of melanoma in melanoma-prone families in which the disease etiology involved major genetic factors, at-risk host pigmentation phenotypes, and environmental exposures.

## Materials and Methods

### Study Population

The details of this family study have been previously described. [28]–[29] In brief, US families with at least two living first degree relatives with a history of invasive melanoma were ascertained through health care professionals or self-referrals. All family members willing to participate in the study underwent a full-body skin examination for phenotypes (type and total number of nevi, extent of freckling, skin complexion, evidence for solar injury, and hair and eye color) and completed risk factor questionnaires for sun-related exposures such as tanning ability. All diagnoses of melanoma were confirmed by histologic review of pathologic material, pathology reports, or death certificates for deceased CMM cases. The study was approved by the National Cancer Institute Clinical Center Institutional Review Board and conducted according to the Declaration of Helsinki. Informed consent was obtained from all participants.

The current study was based on 53 families (23 families segregating *CDKN2A* mutations [*CDKN2A*+] and 30 families without known mutations [*CDKN2A*-]). All study participants were Caucasian. Two controls were selected for each case. The study population for genotyping was comprised of 183 CMM cases and 379 unaffected individuals. TL data was available from a subset of individuals (119 CMM cases and 208 unaffected individuals). The unaffected individuals included 144 unaffected family members and 64 genetically unrelated spouses. Demographic and CMM risk factors did not differ significantly among subjects who were included and not included in the TL analysis (data not shown).

### Telomere Length Measurement

DNA was extracted from whole blood (N = 267) whenever available and from EBV-transformed lymphocytes (N = 60) when whole blood DNA was not available. Quantitative PCR was used to measure telomere length. The average, relative TL was estimated from the ratio of the telomere (*T*) repeat copy number to a single gene copy number (*36B4* gene; *S*), expressed as the *T/S* ratio for each sample using standard curves. All samples for both the telomeres and single-copy gene reactions were performed in triplicate. Three blind replicate samples were interspersed with the samples to assess inter-plate variability. The coefficients of variation (CVs) within triplicates of the telomere assay, single-gene assay, and T/S ratio were 0.87%, 0.65%, and 6.67% respectively. The inter-assay CVs were 0.98%, 1.62%, and 7.92% respectively.

### SNP Genotyping

13 tag SNPs in *TERT* were genotyped at the NCI Core Genotyping Facility (Advanced Technology Center, Gaithersburg, MD; http://snp500cancer.nci.nih.gov) using a custom-designed iSelect Infinium assay (Illumina, www.illumina.com), which included a total of 27,904 tag SNPs that were selected for a variety of cancers. Tag SNPs were selected using a minimum minor allele frequency (MAF) criterion of MAF≥5% based upon HapMap data for Caucasian (CEU) and Yoruban (YRI) samples using Tagzilla, software that implements a tagging algorithm based on pairwise linkage disequilibrium. [30] SNPs within the region spanning 20 kb 5′ of the start of transcription (exon 1) to 10 kb 3′ of the end of the last exon were grouped using a binning threshold of r^2^>0.8 to define a gene/region. When there were multiple transcripts available for genes, only the primary transcript was assessed. SNPs with low completion (<90%) and low concordance (<95%) were excluded. Among 586 genotyped samples, 20 were excluded due to either low completion (<90%, n = 12) or Mendelian inconsistencies (n = 8). Four individuals were further removed from all analyses due to missing CMM status.

### Statistical Analysis

The Wilcoxon-Mann-Whitney test was used to assess whether TL differed significantly between spouse controls and unaffected family members. TL was not significantly different in the two control groups (*P* = 0.63), therefore we combined all controls in the analyses. The Wilcoxon-Mann-Whitney test was used to assess whether TL differed significantly between DNA from whole blood and DNA from EBV-transformed lymphocytes. TL did not differ significantly by DNA source in unaffected individuals (*P* = 0.47) or in CMM cases (*P* = 0.09), however we still adjusted for DNA source in all regression models. We also performed a sensitivity analysis by restricting the evaluation to individuals whose DNA was extracted from whole blood. Spearman correlation was used to evaluate the correlation between TL and age at blood draw, both as continuous variables.

We defined tertiles of TL distribution using cut-points based on the distribution among all unaffected individuals (Short: <0.53; Medium: 0.53–0.72; Long: >0.72) and evaluated the associations between TL and CMM risk factors using a generalized estimating equation approach that accounts for familial correlation in the variance computation, age at blood draw, gender, and DNA source. Conditional logistic regression was used to obtain the odds ratios (OR) and 95% confidence intervals (CIs) for the association between CMM risk and TL, with the shortest telomere tertile used as the reference group. We included age at blood draw, gender, and DNA source in the basic model, and further adjusted for germline *CDKN2A* mutation status, number of nevi, solar injury, and *MC1R* (as a surrogate for pigmentation characteristics) [28] in the final model. Conditioning on families was used to account for family ascertainment and differences in disease prevalence among families. While this approach ignores residual familial correlations among family members, it gives estimates that are attenuated toward the null and is thus conservative [31].

We used conditional logistic regression models to estimate the trend p-value for the association between CMM and each *TERT* SNP, using codominant coding for genotypes (0,1,2) with the homozygote of the common allele as the reference group, and adjusted for age at exam/diagnosis and gender. The associations between TL and genotypes were assessed using a generalized estimating equations (GEE) approach to account for correlation among family members [32], adjusted for age at blood draw, gender, and CMM. ORs and 95% CIs were computed using cumulative logistic regression for ordinal outcomes (PROC GENMOD, SAS 9.1). The working correlation matrix was the independent correlation matrix. We used a Bonferroni correction to account for the number of SNPs and outcomes (CMM and TL) tested, and thus used *P*<0.05/26 (0.002) to define statistical significance. All statistical tests were two-sided and data was analyzed using SAS version 9.1 (SAS Institute, Cary, NC).

## Results

In total, there were 119 CMM cases and 208 unaffected individuals included in the TL analysis. As expected, *CDKN2A* mutations, pale or fair skin type, increased number of nevi, increased number of freckles, solar injury, and *MC1R* variants were significantly associated with CMM risk in these families (Table 1).

**Table 1:** Distribution of age, gender, *CDKN2A*, pigmentation phenotype, and sun exposure variables in 53 melanoma-prone families by CMM status.

	Unaffected Individuals (n = 208)		CMM Cases (n = 119)		
	N	%	N	%	*P* 1
**Age at Blood Draw**					
≤30	41	19.7	16	13.4	
30–40	49	23.6	25	21	
40–50	46	22.1	34	28.6	
50–60	40	19.2	22	18.5	
60+	32	15.4	22	18.5	0.45
**Gender**					
Female	121	58.2	59	49.6	
Male	87	41.8	60	50.4	0.13
***CDKN2A***					
Non-Carrier	178	85.6	59	49.6	
Carrier	30	14.4	60	50.4	<.0001
**Moles**					
0–24	58	29.4	7	6.4	
25–49	37	18.8	14	12.8	
50–99	53	26.9	17	15.6	
100+	49	24.9	71	65.1	<.0001
**Solar injury**					
None/mild	133	67.2	54	50.5	
Moderate	42	21.2	31	29	
Severe	23	11.6	22	20.6	0.01
***MC1R***					
Wild type	35	24.8	7	7.2	
1 nonsynonymous variant	63	44.7	46	47.4	
2 nonsynonymous variants	43	30.5	44	45.4	0.001
**Tanning ability**					
Tan/Little burn	94	51.9	46	46.9	
Burn/Little tan	87	48.1	52	53.1	0.43
**Skin type**					
Dark/medium	64	32.7	18	16.8	
Pale/fair	132	67.3	89	83.2	0.003
**Eye color**					
Black/brown	54	27.8	25	22.9	
Hazel	44	22.7	28	25.7	
Green/gray	16	8.2	11	10.1	
Blue	80	41.2	45	41.3	0.76
**Hair color**					
Black/brown	93	47.4	50	45.9	
Blond brown/light brown	56	28.6	32	29.4	
Blond	28	14.3	11	10.1	
Red	19	9.7	16	14.7	0.47
**Freckles**					
None/few	67	40.6	18	20	
Moderate	40	24.2	29	32.2	
Many	58	35.2	43	47.8	0.004

1 P-values were obtained by comparing CMM cases to unaffected individuals using the chi-square test.

**Figure 1:**
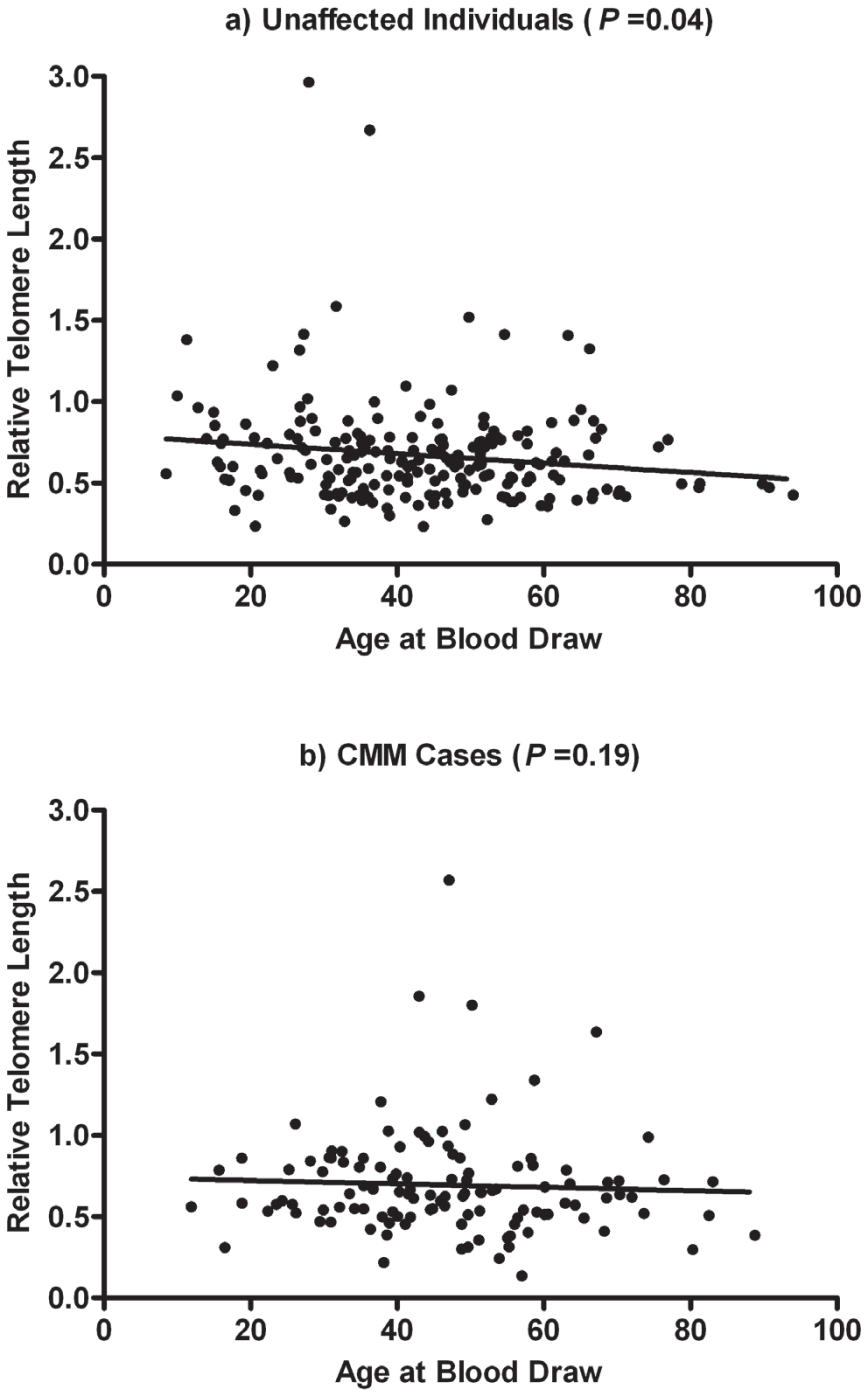
Correlations between relative telomere length and age at blood draw in unaffected individuals and CMM cases. *P* values were obtained from the Spearman correlation test.

As expected, TL was negatively correlated with age at blood draw among both unaffected individuals (r = −0.14) and CMM cases (r = −0.12) (Figure 1). Among unaffected individuals, TL did not differ significantly by any CMM risk factor examined (Table 2). Among CMM cases, shorter telomeres were significantly associated with the presence of moderate or severe solar injury (*P = *0.017) after adjusting for age at blood draw, gender, and DNA source (whole blood or EBV-transformed lymphocytes). Longer telomeres appeared to be associated with increased number of moles in both unaffected and CMM individuals, however, the association was not significant in either phenotype group after the covariate adjustment.

**Table 2:** Distribution of age, gender, *CDKN2A*, pigmentation phenotype, and sun exposure variables in 53 melanoma-prone families by telomere length, stratified by CMM status.

	Unaffected Individuals							CMM Cases						
	Short (n = 70)		Medium (n = 69)		Long (n = 69)			Short (n = 34)		Medium (n = 42)		Long (n = 43)		
	N	%	N	%	N	%	*P* 1	N	%	N	%	N	%	*P* 1
**Age at blood draw**														
≤50	42	30.9	50	36.8	44	32.4		16	21.3	26	34.7	33	44	
50+	28	38.9	19	26.4	25	34.7	0.009	18	40.9	16	36.4	10	22.7	0.03
**Gender**														
Female	38	31.4	45	37.2	38	31.4		14	23.7	21	35.6	24	40.7	
Male	32	36.8	24	27.6	31	35.6	0.77	20	33.3	21	35	19	31.7	0.2
***CDKN2A***														
Non-Carrier	60	33.7	58	32.6	60	33.7		16	27.1	19	32.2	24	40.7	
Carrier	10	33.3	11	36.7	9	30	0.37	18	30	23	38.3	19	31.7	0.09
**Moles**														
0–49	34	35.8	34	35.8	27	28.4		6	28.6	8	38.1	7	33.3	
50+	31	30.4	33	32.4	38	37.3	0.83	22	25	31	35.2	35	39.8	0.61
**Solar injury**														
None/mild	43	32.3	47	35.3	43	32.3		6	11.1	22	40.7	26	48.1	
Moderate/Severe	22	33.8	20	30.8	23	35.4	0.19	22	41.5	16	30.2	15	28.3	0.017
***MC1R***														
Wild type/1 variant	30	30.6	38	38.8	30	30.6		11	20.8	21	39.6	21	39.6	
2 variants	16	37.2	8	18.6	19	44.2	0.61	14	31.8	15	34.1	15	34.1	0.51
**Tanning ability**														
Tan/little burn	30	31.9	37	39.4	27	28.7		10	21.7	15	32.6	21	45.7	
Burn/little tan	28	32.2	28	32.2	31	35.6	0.73	17	32.7	19	36.5	16	30.8	0.08
**Skin type**														
Dark/medium	23	35.9	26	40.6	15	23.4		5	27.8	6	33.3	7	38.9	
Pale/fair	40	30.3	41	31.1	51	38.6	0.15	23	25.8	32	36	34	38.2	0.97
**Eye color**														
Black/brown	15	27.8	24	44.4	15	27.8		9	36	4	16	12	48	
Hazel/green/	49	35	42	30	49	35	0.89	19	22.6	35	41.7	30	35.7	0.65
gray/blue														
**Hair color**														
Black/brown	27	29	35	37.6	31	33.3		11	22	14	28	25	50	
Blond brown/light	28	33.3	29	34.5	27	32.1	0.58	11	25.6	18	41.9	14	32.6	0.26
brown/blond														
Red	9	47.4	3	15.8	7	36.8	0.5	6	37.5	7	43.8	3	18.8	0.09
**Freckles**														
None/few	21	31.3	29	43.3	17	25.4		3	16.7	5	27.8	10	55.6	
Moderate/many	33	33.7	27	27.6	38	38.8	0.06	18	25	28	38.9	26	36.1	0.3

1 P-values were obtained by comparing individuals in the telomere tertiles using a generalized estimating equation accounting for familial correlation in the variance and adjusting for age at blood draw, gender, and DNA source.

Short: <0.53; Medium: 0.53–0.72; Long: >0.72.

Although solar injury was more common among CMM cases than unaffected individuals, CMM cases had longer telomeres (36.1% in the longest tertile) compared to unaffected individuals (33.2%) (Table 3). After adjustment for age at blood draw, gender, DNA source, *CDKN2A* carrier status, number of nevi, solar injury, and *MC1R*, individuals in the longest TL category had close to a 3-fold increase in CMM risk compared to individuals in the shortest TL category (OR = 2.81, 95% CI = 1.02–7.72, *P = *0.04) (Table 3). When *CDKN2A*+ and *CDKN2A*- cases were separately compared to unaffected individuals, we found that the association of longer TL with CMM risk was seen in *CDKN2A*- cases (OR = 3.34, 95% CI = 1.12–10.00, *P = *0.03; comparing longest to shortest TL) but not in *CDKN2A*+ cases (OR = 1.00, 95% CI = 0.42–2.38, *P* = 0.99) (Table 4).

**Table 3:** Association of telomere length with melanoma in 53 melanoma-prone families^1^.

Telomere	Unaffected (n = 208)		CMM (n = 119)		Model 1^3^			Model 2^4^			Model 3^5^		
Tertile^2^	N	%	N	%	OR	95% CI	*P*	OR	95% CI	*P*	OR	95% CI	*P*
1st (short)	70	33.7	34	28.6	Ref			Ref			Ref		
2nd (medium)	69	33.2	42	35.3	1.39	0.74–2.62	0.31	2.42	1.02–5.76	0.05	2.03	0.76–5.44	0.16
3rd (long)	69	33.2	43	36.1	1.38	0.73–2.63	0.33	2.89	1.20–6.94	**0.02**	2.81	1.02–7.72	**0.04**

1 ORs and P-values were obtained from conditional logistic regression with melanoma as the outcome variable.

2 Telomere tertile: Short: <0.53; Medium: 0.53–0.72; Long: >0.72.

3 Model 1: age at blood draw, gender, and DNA source adjustment.

4 Model 2: age at blood draw, gender, DNA source, CDKN2A, and solar injury adjustment.

5 Model 2: age at blood draw, gender, DNA source, CDKN2A, moles, solar injury, and MC1R adjustment.

**Table 4:** Association of telomere length with melanoma in 53 melanoma-prone families, stratified by *CDKN2A* status among cases^1^.

Telomere	Unaffected (n = 208)		CDKN2A+ CMM Cases (n = 60)					CDKN2A- CMM Cases (n = 59)				
Tertile^2^	N	%	N	%	OR	95% CI	*P*	N	%	OR	95% CI	*P*
1st (short)	70	33.7	18	30	Ref			16	27.1	Ref		
2nd (medium)	69	33.2	23	38.3	1.4	0.58–3.36	0.46	19	32.2	2.45	0.81–7.44	0.11
3rd (long)	69	33.2	19	31.7	1	0.42–2.38	0.99	24	40.7	3.34	1.12–10.00	**0.03**

1 ORs and P-values were obtained from conditional logistic regression with melanoma as the outcome variable. Age at blood draw, gender, DNA source, and solar injury adjustment.

2 Telomere tertile: Short: <0.53; Medium: 0.53–0.72; Long: >0.72.

The association between CMM and TL did not change significantly when we restricted the analysis to individuals whose DNA was drawn from whole blood (age and gender adjusted OR = 1.83, 95% CI = 0.85–3.90, *P* = 0.12). Similarly, results did not vary significantly by age at blood draw (<50 vs. ≥50 years), time of blood draw in relation to CMM diagnosis (before vs. after CMM diagnosis), age at CMM diagnosis (<40 vs. ≥40 years), or number of melanomas (single vs. multiple) (data not shown).

We examined whether genetic variants in *TERT* were associated with CMM risk and TL in blood. This analysis included 562 individuals (183 CMM cases and 379 unaffected individuals). Among the 13 tag SNPs genotyped in this analysis, one SNP, rs2735940, was significantly associated with TL (*P* = 0.002) after Bonferroni correction. Another SNP, rs10078761, showed suggestive association with CMM status (*P* = 0.003).

## Discussion

In this exploratory analysis, we evaluated TL in blood in relation to CMM, *CDKN2A* germline mutation status, and CMM risk factors in 53 melanoma-prone families with and without *CDKN2A* mutations. Consistent with a previous report that found that longer telomeres were associated with the development of sporadic CMM [26], we found that longer telomeres were also associated with increased CMM risk in melanoma families, although the association was only seen in cases without *CDKN2A* mutations.

Although extremely short telomeres cause genomic instability and therefore increase cancer risk, senescence induced by telomere shortening places a limit on cell proliferation and is believed to provide a barrier for cancer growth [33]. On the other hand, longer telomeres, which may result from upregulated telomerase when cells reach a critically short TL but do not undergo senescence or apoptosis, may be associated with increased proliferative potential and cancer susceptibility. Constitutive telomerase expression in *TERT*-deficient transgenic mouse models resulted in increased incidence of epidermal tumors and skin wound healing [34]. Therefore, longer telomeres, in combination with decreased cell senescence, may greatly increase the proliferation potential of melanocytes, which leads to an increased propensity for nevi and melanoma. Consistent with a previous study which found that longer telomeres were associated with larger size and number of nevi [27], we found that longer telomeres were associated with an increased number of moles.

Interestingly, the association between longer TL and CMM risk was seen in *CDKN2A*- cases but not in *CDKN2A*+ cases. One possible reason is that melanomas in individuals with *CDKN2A* mutations may develop from telomere-independent mechanisms. Alternatively, the uncontrolled cell proliferation coupled with impaired DNA repair caused by *CDKN2A* mutations may lead to genomic instability and telomere shortening. *CDKN2A*-deficient mice exhibited increased levels of intracellular reactive oxygen species (ROS) in response to UVR [35] and demonstrated reduced ability to process UVR-induced DNA damage [36]. In proliferating cells, telomere DNA can be lost due to the inability of the DNA replication machinery to duplicate the linear DNA ends. Consistent with this hypothesis, a recent study demonstrated that telomere length shortening was significantly associated with hypermethylation of *CDKN2A* promoters in breast cancer [37].

Due to a high content of guanines, telomeres are especially sensitive to damage by oxidative stress [38]. Although the exact mechanism is unknown, telomere shortening is likely caused by oxidative DNA damage and deficiency in DNA repair in telomeric regions [39]. Our observation that moderate or severe solar injury was associated with shorter TL is in line with these findings. It is not clear why the association only occurred among CMM cases but not in unaffected individuals. One possibility is that solar injury reflected the interaction of sun exposure and sun sensitivity, and CMM cases are more likely to have sensitive skin types and be deficient in DNA repair capacities.

Some, but not all of our findings are consistent with those of Bodelon *et al.* based on a Mediterranean population [40]. In that study TL was also significantly associated with age but not with other CMM risk factors among unaffected individuals [40], but in contrast to our results, TL was not associated with CMM risk. The inconsistency is likely due to the differences in the populations, sun exposure, pigmentation characteristics, family history of melanoma, and melanoma phenotypes (usually diagnosed at early-stage in US because of more frequent screening) between the Mediterranean and American populations.

In our study, one SNP in *TERT* (rs2735940) was significantly associated with TL (*P* = 0.002). Another SNP (rs4635969) in *TERT*-*CLPTM1L*, a region that was previously associated with multiple cancers including melanoma [41], showed a suggestive association with TL (*P* = 0.005). A common polymorphism (rs2853669) in *TERT*, which was in complete allelic linkage with the recently identified germline mutation in the promoter of *TERT*, was unfortunately not genotyped in our study and not in LD with any of our genotypted SNPs.

Our study was exploratory due to the limited number of melanoma cases analyzed. In addition, in a small subset of individuals DNA was extracted from EBV-transformed lymphocytes, which could potentially cause bias in TL measurement. However, we adjusted the analysis for DNA source in our logistic regression models. We also restricted the analysis to individuals with DNA extracted from whole blood and the results showed similar patterns. Another limitation was that our families were ascertained primarily through self- or physician-referral, and thus findings may not be generalizable to other familial melanoma sample sets or to sporadic melanoma patients. The strengths of our study include a rich collection of genetic, exposure, clinical, and pigmentation data in melanoma-prone families with and without known *CDKN2A* mutations. We confirmed results from previous studies which found that longer telomeres were associated with CMM risk. Furthermore, our findings suggest that TL in CMM cases might be influenced by multiple mechanisms with opposing directions. Genetic background associated with proliferation potential and at-risk pigmentation phenotypes may predispose CMM cases to longer TL, whereas *CDKN2A* mutations and sun exposure may cause telomere shortening in these individuals. Therefore, using TL alone as a potential biomarker to predict CMM risk may oversimplify the complex role and regulation of telomeres.

## References

[1] Muller HK, Malley RC, McGee HM, Scott DK, Wozniak T, et al (2008) Effect of UV radiation on the neonatal skin immune system- implications for melanoma. Photochem Photobiol 84: 47–54. 18173700 10.1111/j.1751-1097.2007.00246.x

[2] Tucker MA (2009) Melanoma epidemiology. Hematol Oncol Clin North Am 23: 383–395, vii. 10.1016/j.hoc.2009.03.010 PMC3234163 19464592

[3] Goldstein AM, Tucker MA (2001) Genetic epidemiology of cutaneous melanoma: a global perspective. Arch Dermatol 137: 1493–1496. 11708953 10.1001/archderm.137.11.1493

[4] Goldstein AM (2004) Familial melanoma, pancreatic cancer and germline CDKN2A mutations. Hum Mutat 23: 630. 10.1002/humu.9247 15146471

[5] Eliason MJ, Larson AA, Florell SR, Zone JJ, Cannon-Albright LA, et al (2006) Population-based prevalence of CDKN2A mutations in Utah melanoma families. J Invest Dermatol 126: 660–666. 16397522 10.1038/sj.jid.5700094

[6] Bishop DT, Demenais F, Goldstein AM, Bergman W, Bishop JN, et al (2002) Geographical variation in the penetrance of CDKN2A mutations for melanoma. J Natl Cancer Inst 94: 894–903. 12072543 10.1093/jnci/94.12.894

[7] O’Sullivan RJ, Karlseder J (2010) Telomeres: protecting chromosomes against genome instability. Nat Rev Mol Cell Biol 11: 171–181. 20125188 10.1038/nrm2848 PMC2842081

[8] Levy MZ, Allsopp RC, Futcher AB, Greider CW, Harley CB (1992) Telomere end-replication problem and cell aging. J Mol Biol 225: 951–960. 1613801 10.1016/0022-2836(92)90096-3

[9] Kim NW, Piatyszek MA, Prowse KR, Harley CB, West MD, et al (1994) Specific association of human telomerase activity with immortal cells and cancer. Science 266: 2011–2015. 7605428 10.1126/science.7605428

[10] Bodnar AG, Ouellette M, Frolkis M, Holt SE, Chiu CP, et al (1998) Extension of life-span by introduction of telomerase into normal human cells. Science 279: 349–352. 9454332 10.1126/science.279.5349.349

[11] Law MH, Montgomery GW, Brown KM, Martin NG, Mann GJ, et al (2012) Meta-Analysis Combining New and Existing Data Sets Confirms that the TERT-CLPTM1L Locus Influences Melanoma Risk. J Invest Dermatol 132: 485–487. 21993562 10.1038/jid.2011.322 PMC3258346

[12] Horn S, Figl A, Rachakonda PS, Fischer C, Sucker A, et al (2013) TERT promoter mutations in familial and sporadic melanoma. Science 339: 959–961. 23348503 10.1126/science.1230062

[13] Huang FW, Hodis E, Xu MJ, Kryukov GV, Chin L, et al (2013) Highly recurrent TERT promoter mutations in human melanoma. Science 339: 957–959. 23348506 10.1126/science.1229259 PMC4423787

[14] Slagboom PE, Droog S, Boomsma DI (1994) Genetic determination of telomere size in humans: a twin study of three age groups. Am J Hum Genet 55: 876–882. 7977349 PMC1918314

[15] Levy D, Neuhausen SL, Hunt SC, Kimura M, Hwang SJ, et al (2010) Genome-wide association identifies OBFC1 as a locus involved in human leukocyte telomere biology. Proc Natl Acad Sci U S A 107: 9293–9298. 20421499 10.1073/pnas.0911494107 PMC2889047

[16] Andrew T, Aviv A, Falchi M, Surdulescu GL, Gardner JP, et al (2006) Mapping genetic loci that determine leukocyte telomere length in a large sample of unselected female sibling pairs. Am J Hum Genet 78: 480–486. 16400618 10.1086/500052 PMC1380290

[17] Vasa-Nicotera M, Brouilette S, Mangino M, Thompson JR, Braund P, et al (2005) Mapping of a major locus that determines telomere length in humans. Am J Hum Genet 76: 147–151. 15520935 10.1086/426734 PMC1196417

[18] Mangino M, Richards JB, Soranzo N, Zhai G, Aviv A, et al (2009) A genome-wide association study identifies a novel locus on chromosome 18q12.2 influencing white cell telomere length. J Med Genet 46: 451–454. 19359265 10.1136/jmg.2008.064956 PMC2696823

[19] Codd V, Mangino M, van der Harst P, Braund PS, Kaiser M, et al (2010) Common variants near TERC are associated with mean telomere length. Nat Genet 42: 197–199. 20139977 10.1038/ng.532 PMC3773906

[20] Gu J, Chen M, Shete S, Amos CI, Kamat A, et al (2011) A genome-wide association study identifies a locus on chromosome 14q21 as a predictor of leukocyte telomere length and as a marker of susceptibility for bladder cancer. Cancer Prev Res (Phila) 4: 514–521. 21460395 10.1158/1940-6207.CAPR-11-0063 PMC3076128

[21] von Zglinicki T (2002) Oxidative stress shortens telomeres. Trends Biochem Sci 27: 339–344. 12114022 10.1016/s0968-0004(02)02110-2

[22] Wentzensen IM, Mirabello L, Pfeiffer RM, Savage SA (2011) The association of telomere length and cancer: a meta-analysis. Cancer Epidemiol Biomarkers Prev 20: 1238–1250. 21467229 10.1158/1055-9965.EPI-11-0005 PMC3111877

[23] Lan Q, Cawthon R, Shen M, Weinstein SJ, Virtamo J, et al (2009) A prospective study of telomere length measured by monochrome multiplex quantitative PCR and risk of non-Hodgkin lymphoma. Clin Cancer Res 15: 7429–7433. 19934287 10.1158/1078-0432.CCR-09-0845 PMC2787641

[24] Liu J, Yang Y, Zhang H, Zhao S, Liu H, et al (2011) Longer leukocyte telomere length predicts increased risk of hepatitis B virus-related hepatocellular carcinoma: a case-control analysis. Cancer 117: 4247–4256. 21387275 10.1002/cncr.26015

[25] Han J, Qureshi AA, Prescott J, Guo Q, Ye L, et al (2009) A prospective study of telomere length and the risk of skin cancer. J Invest Dermatol 129: 415–421. 18668136 10.1038/jid.2008.238 PMC2632304

[26] Nan H, Du M, De Vivo I, Manson JE, Liu S, et al (2011) Shorter telomeres associate with a reduced risk of melanoma development. Cancer Res 71: 6758–6763. 22028319 10.1158/0008-5472.CAN-11-1988 PMC3206204

[27] Bataille V, Kato BS, Falchi M, Gardner J, Kimura M, et al (2007) Nevus size and number are associated with telomere length and represent potential markers of a decreased senescence in vivo. Cancer Epidemiol Biomarkers Prev 16: 1499–1502. 17627017 10.1158/1055-9965.EPI-07-0152

[28] Goldstein AM, Landi MT, Tsang S, Fraser MC, Munroe DJ, et al (2005) Association of MC1R variants and risk of melanoma in melanoma-prone families with CDKN2A mutations. Cancer Epidemiol Biomarkers Prev 14: 2208–2212. 16172233 10.1158/1055-9965.EPI-05-0321A

[29] Goldstein AM, Struewing JP, Chidambaram A, Fraser MC, Tucker MA (2000) Genotype-phenotype relationships in U.S. melanoma-prone families with CDKN2A and CDK4 mutations. J Natl Cancer Inst 92: 1006–1010. 10861313 10.1093/jnci/92.12.1006

[30] Carlson CS, Eberle MA, Rieder MJ, Yi Q, Kruglyak L, et al (2004) Selecting a maximally informative set of single-nucleotide polymorphisms for association analyses using linkage disequilibrium. Am J Hum Genet 74: 106–120. 14681826 10.1086/381000 PMC1181897

[31] Pfeiffer RM, Gail MH, Pee D (2001) Inference for covariates that accounts for ascertainment and random genetic effects in family studies. Biometrika 88: 16.

[32] Zeger SL, Liang KY (1986) Longitudinal data analysis for discrete and continuous outcomes. Biometrics 42: 121–130. 3719049

[33] Feldser DM, Greider CW (2007) Short telomeres limit tumor progression in vivo by inducing senescence. Cancer Cell 11: 461–469. 17433785 10.1016/j.ccr.2007.02.026 PMC1945093

[34] Gonzalez-Suarez E, Samper E, Ramirez A, Flores JM, Martin-Caballero J, et al (2001) Increased epidermal tumors and increased skin wound healing in transgenic mice overexpressing the catalytic subunit of telomerase, mTERT, in basal keratinocytes. EMBO J 20: 2619–2630. 11387197 10.1093/emboj/20.11.2619 PMC125492

[35] Jenkins NC, Liu T, Cassidy P, Leachman SA, Boucher KM, et al (2011) The p16(INK4A) tumor suppressor regulates cellular oxidative stress. Oncogene 30: 265–274. 20838381 10.1038/onc.2010.419 PMC3003740

[36] Sarkar-Agrawal P, Vergilis I, Sharpless NE, DePinho RA, Runger TM (2004) Impaired processing of DNA photoproducts and ultraviolet hypermutability with loss of p16INK4a or p19ARF. J Natl Cancer Inst 96: 1790–1793. 15572761 10.1093/jnci/djh307

[37] Radpour R, Barekati Z, Haghighi MM, Kohler C, Asadollahi R, et al (2010) Correlation of telomere length shortening with promoter methylation profile of p16/Rb and p53/p21 pathways in breast cancer. Mod Pathol 23: 763–772. 20081803 10.1038/modpathol.2009.195

[38] Kawanishi S, Oikawa S (2004) Mechanism of telomere shortening by oxidative stress. Ann N Y Acad Sci 1019: 278–284. 15247029 10.1196/annals.1297.047

[39] Murnane JP (2012) Telomere dysfunction and chromosome instability. Mutat Res 730: 28–36. 21575645 10.1016/j.mrfmmm.2011.04.008 PMC3178001

[40] Bodelon C, Pfeiffer RM, Bollati V, Debbache J, Calista D, et al (2012) On the interplay of telomeres, nevi and the risk of melanoma. PLoS One 7: e52466. 23300679 10.1371/journal.pone.0052466 PMC3531488

[41] Rafnar T, Sulem P, Stacey SN, Geller F, Gudmundsson J, et al (2009) Sequence variants at the TERT-CLPTM1L locus associate with many cancer types. Nat Genet 41: 221–227. 19151717 10.1038/ng.296 PMC4525478

